# Gene-environment interaction in programming hippocampal plasticity: focus on adult neurogenesis

**DOI:** 10.3389/fnmol.2015.00041

**Published:** 2015-08-04

**Authors:** Muriel Koehl

**Affiliations:** ^1^INSERM U862, Magendie Neurocenter, Neurogenesis and Pathophysiology Group, Institut F. MagendieBordeaux Cedex, France; ^2^Université de BordeauxBordeaux, France

**Keywords:** genetics, environment, life events, hippocampus, neurogenesis, strain

## Abstract

Interactions between genes and environment are a critical feature of development and both contribute to shape individuality. They are at the core of vulnerability resiliency for mental illnesses. During the early postnatal period, several brain structures involved in cognitive and emotional processing, such as the hippocampus, still develop and it is likely that interferences with this neuronal development, which is genetically determined, might lead to long-lasting structural and functional consequences and increase the risk of developing psychopathology. One particular target is adult neurogenesis, which is involved in the regulation of cognitive and emotional processes. Insights into the dynamic interplay between genes and environmental factors in setting up individual rates of neurogenesis have come from laboratory studies exploring experience-dependent changes in adult neurogenesis as a function of individual’s genetic makeup. These studies have implications for our understanding of the mechanisms regulating adult neurogenesis, which could constitute a link between environmental challenges and psychopathology.

## Introduction

Clinical studies related both to neurodegenerative and psychiatric illnesses indicate that gene-environment interactions play an important part in the expression or the etiology of these diseases. Thus, examples of genetic pathologies that are differently expressed according to the environment (Migliore and Coppedè, 2009), such as huntington disease (Mo et al., 2015), Alzheimer disease (Swaminathan and Jicha, 2014) or Parkinson disease (Kieburtz and Wunderle, 2013) are now available, and both epidemiologic and clinical reports point to an important role of life events in precipitating mental disease (Caspi et al., 2003; Caspi and Moffitt, 2006).

In this context, because they act during critical developmental periods, environmental factors at play during early life have definitive “reprogramming” effects and gene-early life environmental factors were shown to interact to define vulnerability or resiliency for mental illnesses in adulthood (Heim et al., 2010). During the early postnatal period, several brain structures involved in cognitive and emotional processing, such as the hippocampus, still develop (Schlessinger et al., 1975; Altman and Bayer, 1990). It is thus very likely that interferences with this neuronal development, which is genetically determined, might lead to long-lasting structural and functional consequences and increase the risk of developing psychopathology. A particular feature of the dentate gyrus (DG) is the ability to produce new neurons in adulthood, a process that was shown to contribute to specialized functions such as learning and memory (Koehl and Abrous, 2011) and control of anxiety and mood states (Revest et al., 2009), hence the interest in the different factors involved in its regulation.

Although a central issue in psychiatry is to determine how the gene-environment interactions can explain individual differences in vulnerability/resiliency to psychopathology, in the context of adult neurogenesis, genetic and environmental factors are often studied alone in approaches that attempt to reduce the experimental variation by focusing on one strain. Nevertheless a few examples of the literature have tackled the importance of this interaction, which will be the focus of this review after a brief presentation of methods in gene-environment studies and of adult neurogenesis.

## Importance of Gene-Environment Interplay in Shaping Phenotype: A Historical Overview

Our current scientific opinion regarding the origin of individual differences in personality, aptitudes, and behavioral traits in general, is that neither genetic nor environmental differences are solely responsible for producing phenotypic variation, and that virtually all traits result from the joint influence of genetic and environmental factors. However, this consensual view took time and effort to prevail, and heated nature vs. nurture debates, which assumed that variation in a trait is primarily due to either genetic or environmental differences, were opposing scientists in the 1940’s and 1950’s.

The original compromise and recognition that both genes and environmental factors could explain a part of the phenotypic variance led to a simple equation: Phenotypic variance (V_P_) = Genetic variance (V_G_) + Environmental variance (V_E_), and two major approaches have been developed and used by behavior geneticist to analyze the respective contribution of both factors: the first one consists in studying the effects of environment alone by holding the genetic make-up of the individual constant (V_P_ = V_E_). In essence this consists in placing individuals of the same genotype in different environments and comparing their phenotypic response. This approach, which can be easily developed in humans—comparing twins raised in different families (Bouchard et al., 1990)—, benefits from the existence of inbred strains of mice in which all individuals are isogenic, allowing to directly test the influence of environmental impact. As one can imagine, the second approach consists in studying the effects of genes alone by holding environment constant (V_P_ = V_G_). Typical example is the use of knock-outs (KO), mainly in mice, or selective breeding of rats for a specific trait that is selected and enriched across generations. One of the first influential studies addressing this aspect is a classic selective breeding study in which Tryon (1942) selected rats for their ability to find their way in a maze. He mated the animals that made the fewest errors (maze bright) together and the ones that made the most errors (maze dull) together. Then he mated the most similar offspring for 21 generations, and after seven generations, he had already developed two genetically different lines of rats (maze bright and maze dull). This study was highly influential in the field of psychology for showing that specific behavioral traits may be hereditary.

Finally, with the recognition that phenotypic variance was sometimes not explained by the simple additive contribution of genetic and environmental factors, a third term V_GE_, which measures how much of this variance is due to an interaction between the genotype and the environment, has been introduced. It can be experimentally approached by comparing the effects of different environments on different genotypes. Among the first examples of this approach, Cooper and Zubek compared the performances of Tyron’s rats when raised in either a restricted (an empty cage with gray walls) or an enriched environment (EE; a cage with designs on its walls that contained objects such as ramps, mirrors, swings, balls, slides, and tunnels). They observed a drastic decrease in the performances of bright rats when raised in the restricted environment, which did not affect the already bad performances of dull rats, and an improvement of the performances of dull rats that reached bright rats levels when raised in an EE. With regard to these results, Cooper and Zubek argued that heredity and environment always interact to produce final behavior (Cooper and Zubek, 1958).

Following up this seminal experiment, many studies have been conducted along that line, among which I selected two as they have been, to my point of view, highly influential in the field. In the first one, Crabbe et al. (1999) compared the behavior of mice from different strains in different labs, but using the exact same protocols and apparatus. He showed as expected that genotype was a highly significant parameter for all behaviors studied, accounting for 30–80% of the total variability, and that several documented strain differences were confirmed. However he also found that despite standardization, there were systematic differences across labs, and that for some tests, the magnitude of genetic differences depended upon the testing lab. Altogether the authors highlighted that given the importance of the gene-environment interactions revealed in their study, “***experiments characterizing mutants may yield results that are idiosyncratic to a particular laboratory***”. The second study performed by Francis and colleagues aimed at distinguishing genetic and early environment contributions to the expression of adult behavior in mice. To this end, they used classical fostering approaches, and investigated the effects of prenatal (embryo transfer) and postnatal (cross-fostering) environments in two strains of inbred mice with profound and reliable differences in behavior, namely C57Bl/6J and Balb/cJ mice. They found that C57Bl/6J mice that developed in a Balb/cJ uterus and were reared by a Balb/cJ mother displayed a Balb/cJ phenotype in most behavior tested (exploration, anxiety, spatial learning), while C57Bl/6J mice exposed only to a uterine or a postanatal Balb/cJ environment did not change their phenotype. For the first time, this dataset emphasized the crucial interaction of pre- and post-natal environments in shaping the development of selected behaviors, and further indicated that prenatal environment may prime the developing pup to respond to postnatal environment—most certainly cues delivered by maternal care—in a way that would allow a strain-specific behavior to develop independently of genotype (Francis et al., 2003). Although this study very elegantly demonstrated the importance of gene-environment interactions, one can regret that the same experimental design was not applied to Balb/cJ mice as it is highly probable that these environmental influences can act only within a genetic constraint and that different sensitivities can be expected depending on genetic background, a hypothesis that remains to be tested as of today.

## The Biology of Adult Hippocampal Neurogenesis

Neurogenesis was until quite recently thought to be specifically an ontogenetic aspect of Central Nervous System (CNS) development. However during the last 20 years there has been unequivocal evidence that new neurons are produced in adulthood, not only in lower vertebrates, but also in mammalian species including humans. Two discrete zones of the adult brain have been described as neurogenic: the olfactory bulb and the DG of the hippocampal formation, the focus of this review.

**Figure 1:**
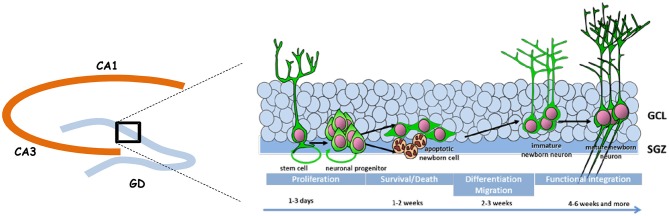
**Schematic representation of adult neurogenesis.** New neurons that integrate the granule cell layer (GCL) of the dentate gyrus (DG) originate from stem and precursor cells located in the subgranular zone (SGZ). In the course of their maturation they exhibit specific properties that confers them a unique behavioral function.

Adult neurogenesis is permitted by the maintenance in the DG of a neurogenic niche that derives from the tertiary dentate matrix mostly active during the early postnatal period and from which most granule cells of the DG are originating (for review, see Lajud and Torner, 2015). This neurogenic niche is composed of neural stem cells and progenitors that produce immature new neurons; these migrate locally into the granule cell layer (GCL) to become dentate granule cells. Adult neurogenesis appears to recapitulate the complete process of neuronal development, ranging from neural progenitor activation and fate determination, to differentiation, migration, and axonal and dendritic development of newborn neurons, to synapse formation and functional integration into the existing neural circuitry (Figure 1).

Adult neurogenesis is tightly regulated by the local environment, the so called “neurogenic niche” that is composed of the extracellular matrix and various cell types, including astroglia, ependymal cells, endothelial cells, immature progeny of adult neural stem cells and mature neurons within the local circuitry. The niche is also a target of many physiological, pathological and pharmacological stimuli that regulate adult neurogenesis.

## Gene-Environment Interplay in Controlling Adult Hippocampal Neurogenesis

Both genetic and environmental contributions to adult neurogenesis have been, and are still, widely studied. Thus in the course of the last 20 years, direct strain comparisons and analysis of the genetic contribution to the variance observed in adult neurogenesis has shown that genetic variation among strains accounted for differences in all aspects of hippocampal neurogenesis, proliferation, survival and differentiation, as well as overall hippocampal volume and total cell numbers (Kempermann et al., 1997a), and that 85% of the variance in neurogenesis between strains could be accounted for by different cell-survival rates while proliferation was only a mild predictor of neurogenesis (Kempermann et al., 2006). Many genes have been singled out that control the different steps of neurogenesis, constituting a molecular signature (Miller et al., 2013), and we refer readers to the MANGO database that lists the different genes involved in controlling non pathological neurogenesis (Overall et al., 2012). In regard to these data, the prevailing view is that adult neurogenesis is a complex phenomenon that is likely to be controlled by the interaction of several regulatory loci involving many genes, and not one master regulatory locus acting as a switch to turn neurogenesis “on” or “off”. In parallel, many studies have shown that different life events were capable of regulating or controlling neurogenesis, and both adult experience (Opendak and Gould, 2015) and early life events (for review, see Fenoglio et al., 2006; Korosi et al., 2012; Lucassen et al., 2013; Hoeijmakers et al., 2014) largely contribute to its levels, the impact of early life events appearing more pervasive and permanent, certainly for they act during developmental periods.

On the opposite, studies carefully controlling both genetic and environmental factors in order to assess the importance of their interactions in determining adult neurogenesis are scarce. The most important ones are those related to the impact of early life environment for the known role of factors acting during this period in shaping phenotypes, and I will focus on these studies. However, because there are only a few of them, and because much information can be gained from the analysis of gene-later life environment interactions, I will also detail these latter studies.

### Impact of Early Life Environment

Many studies have reported that prenatal stress (PS) plays a very influential role in determining the rate of adult neurogenesis (Lemaire et al., 2000; Ortega-Martínez, 2015), but very few have visited this question in relation to genetic background. To the best of our knowledge, only one study by Lucassen and colleagues addressed this issue and compared the impact of PS on early postnatal neurogenesis between rats genetically divergent as issued from a selective breeding for high- and low-anxiety-related behavior, high anxiety bred (HAB) and low anxiety bred (LAB) respectively (Lucassen et al., 2009). As hypothesized, the effects of PS were found to be dependent on the genetic background of the mother and the survival rate of newborn cells and the number of immature neurons doublecortin immunoreactive (DCX-IR) were significantly altered in offspring of stressed HAB rats compared to control HAB but not in offspring of LAB rats. Authors also tested whether the different sensitivity of HAB and LAB rats may be related to placental 11β-HSD2 levels as their variations were found to represent a physiological link between environmental challenges to the pregnant female and programming of the fetal brain, and found increased levels of 11β-HSD2 activity in PS-LAB, suggesting it may have a protecting effect against the programming consequences of PS.

The impact of the postnatal period has been addressed in mice, a gold standard model for this type of analysis because they offer a large variety of genetic backgrounds, but again these studies are scarce. Thus neurogenesis was found to be insensitive to maternal separation (Navailles et al., 2008) in mice from two strains known to differ in their stress reactivity and their levels of neurogenesis: C57Bl/6J, which exhibit a strong resistance to stress—albeit not to early life events (Francis et al., 2003)—, and high levels of neurogenesis, and Balb/cJ, which are known for their liability to stress and low levels of neurogenesis (Kempermann et al., 1997a). Interestingly, this postnatal manipulation strongly inhibits neurogenesis in outbred rats (Mirescu et al., 2004; Oomen et al., 2010), thus raising the question of strain sensitivity to postnatal developmental forces. In order to address this question, we have developed a model allowing to single out the influence of the genetic make-up of the individual on the outcome of maternal care on adult neurogenesis. We selected C57Bl/6J mice and DBA/2J mice for their differences in baseline neurogenesis (Kempermann and Gage, 2002) as well as in their sensitivity to environmental experiences, DBA/2J mice appearing more vulnerable than C57Bl/6J mice (Cabib et al., 2000). Mice from both strains were raised by mothers of non-related strains that displayed high and low levels of maternal care, independently of the strain of fostered pups. This experimental design allowed us to compare neurogenesis in mice from two different genetic backgrounds exposed to the same environmental influences. We reported that maternal care had a major impact on neurogenesis—targeting both the number of immature newborn cells and their morphology—exclusively in DBA/2J mice, thus genetically prone to respond to these influences (Koehl et al., 2012). Interestingly, we had previously reported that DBA/2J mice raised in a high maternal care environment exhibited an anhedonic endophenotype (van der Veen et al., 2008) that could be linked to the delayed maturation of immature granule cells, while C57Bl/6J mice showed resiliency to both the neuroanatomical and behavioral consequences of variations in maternal care. This is particularly relevant to the human condition as most psychiatric theories relate the development of depression to a disruption of mother-infant interactions in certain vulnerable individuals (Rutter et al., 2006), and as neurogenesis has been linked to the effects of antidepressants (see below).

Finally, taken together with results of Navailles et al. (2008), this study confirms that the setpoint for adult neurogenesis in C57Bl/6J mice appears independent of the postnatal environment and a rapid analysis would lead to the conclusion that this setpoint is determined prenatally for C57Bl/6J mice and postnatally for DBA/2J mice, while it may not be influenced by early environment in Balb/cJ mice. However, because an interaction of both pre and postnatal environments was found to be necessary to influence behavior in adult C57bl/6J mice (Francis et al., 2003), and because some postnatal manipulations, such as handling, have no net impact on neurogenesis but can counteract the impact of PS in outbred rats (Lemaire et al., 2006), we cannot exclude that neurogenesis in C57Bl/6J and Balb/cJ mice is determined by a combination of pre and postnatal factors that need to play in synergy.

### Impact of Enriched Environment and Voluntary Exercise

Early life events are not the only shaping factors of adult neurogenesis that have been studied in the context of gene-environment and researchers have showed a lot of interest for the impact of voluntary exercise, one of the most potent adult regulator of neurogenesis. The first evidences of genetic differences in response to complex environment in adulthood are indirect and emerge from comparing results from different studies. Thus in his seminal paper published in 1997 in Nature, Gerd Kempermann reported that upbringing weanling female mice from the C57Bl/6J strain in an EE for 40 days elicited a robust increase in the survival of newly born cells while it had little or no influence on their proliferative activity (Kempermann et al., 1997b). In parallel, he also reported the existence of a drastic variation in baseline levels of precursor proliferation among different mouse strains (Kempermann et al., 1997a), with mice from the 129/SvJ strain having extremely low levels of adult neurogenesis compared to most other inbred strains, and in particular C57Bl/6Jmice. Using the exact same environmental procedure as in his earlier study, he then analyzed the impact of EE in these mice, and reported that in contrast to C57Bl/6J mice, it increased proliferation of progenitor cells as well as the net number of surviving cells, although it actually decreased their survival rate, in 129/SvJ mice. Although he did not run a direct comparison of the two strains responsiveness, he found that the net neurogenic effect of EE was similar in C57Bl/6J and 129Sv/J mice, but that the mechanisms involved differed. This is consistent with the fact that proliferation, survival and differentiation of progenitor cells and their progeny are each separately influenced by inheritable traits and not uniformly upregulated in response to environmental stimulation (Kempermann et al., 1998).

Following up on this seminal discovery, Van Praag and colleagues attempted to separate components of the EE that could explain its pro-survival effect and focused on physical activity (van Praag et al., 1999). They showed in the same mouse line (C57Bl/6J) that voluntary exercise in a running wheel strongly increased cell survival, albeit to a lower extent than EE. It also strongly stimulated cell proliferation, an effect not observed in the EE condition in this strain. Since then the sensitivity of divergent inbred strains of mice to voluntary exercise has been complemented by direct strain comparisons. Thus a study comparing the neurogenic response to exercise in 12 isogenic mouse strains reported that although exercise increased neurogenesis in all 12 strains tested, the magnitude of the effect depended on genotype (Clark et al., 2011). In particular, C57Bl/6J mice, which are the most widely used in studies related to exercise that do not take into consideration genetic influences, was found to be the least responsive strain. Furthermore, a significant percentage of the strain variation in exercise-induced neurogenesis could be accounted for by the distance the animals ran, but removing this factor did not abolish the strain effect, indicating that the quantitative increase in total number of new neurons resulting from exercise differs between strains with some strains showing relatively more new neurons for the same amount of running as compared to others. For example, 129-related strains were found to display a very strong relationship between level of wheel running and number of new neurons, C57Bl/6J mice were intermediate, and DBA/2J showed near zero or negative correlations, indicating no relationship between wheel running and number of new neurons (Merritt and Rhodes, 2015). The same type of observation was reported in another study specifically comparing the effects of exercise on C57Bl/6J and DBA/2J mice, which even reported that although running has pro-proliferative and pro-survival consequences in C57Bl/6J mice, it has only a delayed pro-proliferative effect in DBA/2Jmice that is not correlated to the amount of running (Overall et al., 2013). Altogether this discrepancy between the two strains comforts indications that proliferation and survival programs are mediated by different mechanisms (Kempermann et al., 2006). Interestingly it was also reported that genetic variation in neurogenesis under standard housing conditions was unrelated to running levels of neurogenesis, suggesting that different genes influence variation in adult hippocampal neurogenesis under sedentary vs. runner conditions. Authors further analyzed heritability (the proportion of differences of a trait among individuals of a population that are due to genetic differences), and reported a heritability score of 0.53 for sedentary and 0.33 for runner mice, all strains confounded (Clark et al., 2011).

### Impact of Pharmacological Treatment: Example of Fluoxetine

Similarly to the impact of EE and voluntary exercise, first evidences of strain differences in neurogenic sensitivity to pharmacological treatments are indirect. Thus after pro-proliferative consequences of treatments with serotonin-specific reuptake inhibitors (SSRI) antidepressants such as fluoxetine were reported (Malberg et al., 2000), Santarelli and colleagues further analyzed the neurogenic consequences of this treatment and reported an increase in cell proliferation (Santarelli et al., 2003) as well as a stimulation of dendritic maturation of newborn cells (Wang et al., 2008) in 129SvEv mice. They also showed that disrupting the neurogenic effects of fluoxetine by irradiation disrupted its behavioral consequences, thus suggesting that neurogenesis is required for the behavioral effects of antidepressants (Santarelli et al., 2003).

Following this seminal paper, studies from the same group and others brought controversies in the conclusion. Thus the same authors administered fluoxetine to Balb/cJ mice, a strain prone to anxiety that is used to develop behavioral tests of antidepressants sensitive to chronic but not acute treatment, as observed in humans. They reported that irradiation did not prevent the behavioral effects of fluoxetine while it dramatically reduced adult neurogenesis by ablating progenitor cells. As this result was in opposition to their previous observations, they checked many alternative explanation to conclude that the differences in response to chronic fluoxetine were linked to the strain of mice used and not the tests and paradigms used (Holick et al., 2008).

Although this seems conceivable, a thorough reading of their first study indicated that in order to reach this conclusion, they had also used mice from the Balb/cJ strain and reported the same global observations: irradiation, which blocks cell proliferation, prevented the action of fluoxetine on behavioral responses to chronic unpredictable stress, leaving the question of Balb/cJ mice neurogenic sensitivity to fluoxetine unsolved. However, contemporaneous papers confirmed that although Balb/cJ mice respond behaviorally to chronic fluoxetine treatment, they do not display any increase in neurogenesis (Huang et al., 2008), confirming the assumption of strain differences in sensitivity to fluoxetine, and indicating that neurogenesis may not always be required for the behavioral effects of fluoxetine, which appear to be strain dependent.

Confirming these indirect conclusions, a study by Navailles and collaborators directly compared neurogenic responses of Balb/cJ and C57Bl/6J mice to fluoxetine treatment during adolescence and found that although both strains reacted with an initial increase in cell proliferation, the expected increase in 2 weeks old cells observed in C57Bl/6J was totally absent in Balb/cJ mice, suggesting that the newborn cells did not survive in this strain (Navailles et al., 2008).

Altogether, although they have not been exploited in this direction so far, these strain differences are extremely interesting if one considers that responsiveness to antidepressants is highly variable among patients and that this strain difference certainly has clinical relevance. We thus propose to complete Holick and colleagues statement that “***it is imperative that future studies utilize rodent behavioral models sensitive to chronic antidepressant treatment to dissect which of these neural changes play a causal role in their behavioral effects***” (Holick et al., 2008) by adding that it is also imperative to thoroughly compare sensitive and insensitive strains to dissect the neural changes induced by treatment that are specifically relevant for their behavioral consequences.

## Conclusion

**Figure 2:**
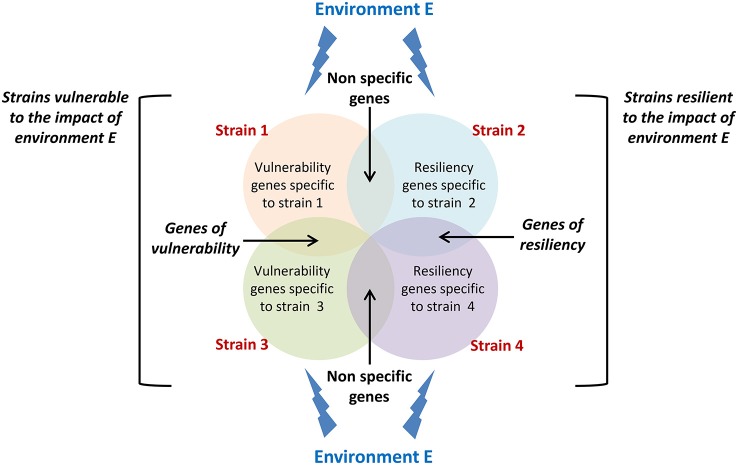
**Conceptual framework for isolating vulnerability and resiliency genes.** Circles represent genes which expression changes in response to a specific environment E in different strains displaying either vulnerability (strain 1 and strain 3) or resiliency (strain 2 and strain 4) to this environment. Genes that expression varies in both vulnerable and resilient strains cannot sustain vulnerability or resiliency and should be disregarded. Genes that expression varies in both vulnerable strains and not in resilient strains are potential genes sustaining vulnerability to environment E. The same applies to resiliency genes.

The latent idea behind gene-environment interaction studies is to identify molecular or neurobiological factors common to high responding strains and uncommon to non-responding strains that are capable of governing the genesis of new neurons in the adult hippocampus. As this field of research is still at its premises, studies so far have mainly demonstrated the existence of a complex interaction between genetic constraints and life events but none of them has yet identified the mechanisms involved. A proposed setup for future studies would be to analyze changes in gene expression in strains showing sensitivity and resistance to a specific environmental stimulation (Figure 2) in order to isolate those changes that specifically support vulnerability and resiliency of adult neurogenesis to environmental challenges.

Overall, data presented in this review thus indicate that in addition to genes that have been isolated for their control of the different phases of neurogenesis, the regulation of adult neurogenesis is also governed by a set of genes that confer to an individual an increased sensitivity or a certain resistance to life events. Isolating these genes (Figure 2), the mechanisms by which environmental factors can affect their expression, and the mechanisms by which they regulate adult neurogenesis will constitute major steps in our understanding of individual differences in adult neuroplasticity.

## Conflict of Interest Statement

The author declares that the research was conducted in the absence of any commercial or financial relationships that could be construed as a potential conflict of interest.
